# *Odontomachus davidsoni* sp. nov. (Hymenoptera, Formicidae), a new conspicuous trap-jaw ant from Ecuador

**DOI:** 10.3897/zookeys.948.48701

**Published:** 2020-07-13

**Authors:** Philipp O. Hoenle, John E. Lattke, David A. Donoso, Christoph von Beeren, Michael Heethoff, Sebastian Schmelzle, Adriana Argoti, Luis Camacho, Bernhard Ströbel, Nico Blüthgen

**Affiliations:** 1 Ecological Networks, Department of Biology, Technical University of Darmstadt, Darmstadt, Germany; 2 Departamento de Zoologia, Universidade Federal do Paraná, Curitiba, Brazil; 3 Departamento de Biología, Escuela Politécnica Nacional, Quito, Ecuador; 4 Centro de Investigación de la Biodiversidad y Cambio Climático, Universidad Tecnológica Indoamérica, Quito EC170103, Ecuador; 5 Escuela de Ciencias Biológicas, Pontificia Universidad Católica del Ecuador, Quito, Ecuador; 6 Department of Zoology, University of British Columbia,Vancouver, Canada; 7 University of Applied Sciences, Darmstadt, Germany

**Keywords:** 3D scan, DNA barcoding, DISC3D, integrative taxonomy, Northwest Ecuador, Ponerinae, tropical forest

## Abstract

One of the largest species in its genus, *Odontomachus davidsoni* Hoenle, Lattke & Donoso, **sp. nov.** is described from workers and queens collected at lowland forests in the Chocó-Darién bioregion in coastal Ecuador. The workers are characterized by their uniform red coloration, their large size (16–18 mm body length), and their frontal head striation that reaches the occipital margin. DNA barcodes (COI) and high resolution 2D images of the type material are provided, as well as an updated key for the Neotropical species of *Odontomachus*. In addition, a three-dimensional digital model of the worker holotype and a paratype queen scanned with DISC3D based on photogrammetry is presented, for the first time in a species description. Findings of large and conspicuous new species are uncommon around the world and suggest that these Ecuadorian rainforests may conceal many more natural treasures that deserve conservation.

## Abstract (es)

Describimos una especie nueva, entre las más grandes conocidas del género *Odontomachus*. La nueva especie, *Odontomachus davidsoni* Hoenle, Lattke & Donoso, sp. nov., es descrita a partir de obreras y reinas recolectadas en bosques de tierras bajas en la bioregión Chocó-Darién de la costa del Ecuador. Las obreras se caracterizan por su coloración rojiza uniforme, su grande tamaño (largo del cuerpo 16–18 mm), y la estriación del frente cefálico que alcanza el margen occipital. Proveemos códigos de barras de DNA (COI) e imágenes 2D de alta resolución para el material tipo y así como una guía de identificación actualizada para las especies neotropicales del género *Odontomachus*. Por primera vez en una descripción de especies, se proveen imágenes 3D de un escáner fotogramétrico DISC3D. Los hallazgos de especies grandes y conspicuas son poco comunes alrededor del mundo y sugieren que estos bosques lluviosos ecuatorianos pueden contener muchos más tesoros naturales que merecen ser conservados.

## Introduction

Members of the genus *Odontomachus* Latreille, 1804 are among the most conspicuous and recognizable ants of the subfamily Ponerinae. All members of the genus exhibit trap jaws, a character shared with the sister genus *Anochetus* Mayr, 1861 (Schmidt and Shattuck 2014) and several other unrelated genera (Larabee and Suarez 2014). Trap jaws are long, slender mandibles that are mainly used for predation and secondarily in some taxa to catapult the ant into the air as a predator escape response (Larabee and Suarez 2015). Trap jaws function via a special clamping mechanism with the mandibles opening at a 180-degree angle and snap shut at high speeds upon physical contact with mechanosensory hairs (Gronenberg et al. 1993; Gronenberg 1995). *Odontomachus* typically comprises medium- to large-sized ants with a pantropical distribution including subtropical regions. The genus currently contains a total of 72 species (Bolton 2018), with the highest diversity found in the Neotropics and South-East Asia (Larabee et al 2016; Guenard et al 2017; Matos-Maraví et al. 2018).

Ecuador, with at least 18 *Odontomachus* species (Salazar et al. 2015), is among the countries showing the highest species diversity recorded for this genus. In general, Ecuadorian biodiversity is comparatively high, because the country comprises three vastly distinct bioregions: the Amazon basin in East Ecuador, the Chocó-Darién bioregion in the North-West, and the Tumbesian drylands in the South of the country which are divided by the Andes, one of the highest mountain chains in the world (Salazar and Donoso 2013; Salazar et al. 2015). While ants of the Ecuadorian Amazon have received some attention (e.g., Donoso et al. 2009; Wilkie et al. 2010), ant research is severely lacking in the highly threatened areas of the Chocó-Darién (Donoso and Ramón 2009; Donoso 2017) and in the southern drylands (Dominguez et al. 2016; Lattke et al. 2016). Notably, the Chocó-Darién is home for several large and rare *Odontomachus* species, including *O. mormo* Brown, 1976, and *O. cornutus* Stitz, 1933, both of which are only known from few collections (Rodriguez 2008). Since Brown’s (1976) global revision of the genus, the taxonomy of the New World species has remained relatively stable, with one additional species described in the United States (Deyrup & Cover 2004) and none for the Neotropics except for *O. scalptus* Brown, 1978, from Tena, in the Ecuadorian Amazon.

We describe a surprisingly large new species, i.e., *O. davidsoni* sp. nov., from the Ecuadorian Chocó-Darién region that rivals in size *O. mormo*, previously considered the largest species of New World *Odontomachus*. We use morphological and genetic analyses to distinguish it from other *Odontomachus* species and use, for the first time in insect taxonomy, textured 3D-models to present a digital version of the holotype, a queen and several other specimens.

## Materials and methods

### Sampling and geographic origin

Specimens of *O. davidsoni* were collected and observed in field trips to the Reserva Río Canandé in Ecuador (Esmeraldas Province) from February 2018 to April 2019. We searched for specimens by walking through the forest and examining the vegetation and recently fallen trees. Alate queens were collected with light traps directly located at the Ecolodge of the Reserve. We collected exclusively by hand, and specimens were preserved in vials containing 96% ethanol. Photographs of living specimens were taken with a Nikon D5300 camera body (Nikon Corp., Tokio, JP) and a Laowa 60mm f2.8 2× macro lens (Venus Optics, Hefei, China). The Ministerio de Ambiente de Ecuador issued the permits for collection (MAE-DNB-CM-2017-0068) and exportation (41-2018-EXP-CM-FAU-DNB/MA and 144-2019-EXP-CM-FAU-DNB/MA).

### Photogrammetry

The worker holotype, two worker paratypes, and one paratype queen were mounted with water-soluble insect glue on the tip of an insect needle and imaged using the Darmstadt Insect Scanner (DISC3D, Ströbel et al. 2018). Specimens were imaged with extended depth of field (EDOF) using calibrated stacks of 19 images with 4 megapixels (Ströbel et al. 2018). EDOF images were taken from 398 viewing angles (in total 7562 images were recorded) and used for photogrammetric reconstruction and texturing in PhotoScan Professional 1.4.5. (Agisoft LLC, St. Petersburg, Russia) with the highest quality settings and visibility consistent mesh generation. Polygons corresponding to the insect pin were removed from the resulting mesh and the mesh was slightly smoothed (with a factor of 0.5). In addition, the holotype and queen models were textured using the “average” option resulting in 5000 × 5000 pixel texture maps. Textures were sharpened and cleaned in Adobe Photoshop CS6 13.0 (Adobe Inc., San Jose, CA, USA). We furthermore provide videos with added light effects created with Blender (Blender Foundation; Amsterdam, NL; Suppl. materials 1, 2).

Additional stacking pictures were taken with a Canon EOS 7D with a MPE 65mm lens (Canon, Tokyo, Japan) and a Keyence VHX-5000 (Keyence Deutschland BmH, Neu-Isenburg, Germany) with a Z20 lens. Stacking pictures were assembled with Helicon Focus Version 7 (Helicon Soft Ltd., Kharkiv, Ukraine) software, and further edited with Adobe Photoshop CS6 13.0 (Adobe Inc., San Kaso, CA, USA).

### Morphological data

Morphological measurements of three workers (the holotype and two paratypes) and one alate queen were performed using 3D-models embedded in PDF files obtained with DISC3D. The measurements obtained through such models are more precise and reproducible than traditional ocular micrometer measurements, partly since parallax errors are avoided (see Ströbel et al. 2018). Measurements were taken with Adobe Acrobat Reader DC (version 11.0.23; Adobe Inc., San Jose, CA, USA) following the definitions described in MacGown et al. (2014) and Brown (1976), with the exception of head and petiole height measurements (see definitions below). As Brown (1976) already recognized, heads of *Anochetus* and *Odontomachus* can have a relatively trapezoid or more rectangular shape in frontal view, which is a reliable indicator to help differentiate between species. To discern between these shapes, we included the head ocular width (HoW – synonymous with HW in other studies, e.g., MacGown et al. 2014) and head vertexal width (HvW, see below). We measured two paratype workers with a binocular with measuring eyepiece only (see Suppl. material 3). The morphological values given in the species description however solely derive from measurements of the 3D scan of workers (*N* = 3) and queen (*N* = 1) for reasons of consistency. All measurements are given in millimeters. Additional to the measurements we give a detailed morphological description of *Odontomachus davidsoni* sp. nov. The morphological terminology, including those of surface sculpturing, is based on definitions found in Wilson (1955), Harris (1979) and Keller (2011).

### Measurement definitions

All measurements are given in millimeter.

**CI** Cephalic index. HW/HL × 100.

**EL** Eye length. Maximum length of eye as measured normally in oblique view of the head to show full surface of eye.

**FL** Femur length. Maximum length of hind femur.

**HL** Head length. Maximum length of head in full-face view, excluding mandibles, measured from anteriormost point of clypeal margin to midpoint of a line across the posterior margin.

**HoW** Head ocular width. Maximum width of head at ocular prominence in full-face view, measured in the same plane as HL.

**HvW** Head vertexal width. Width of head at vertex in full-face view, measured in the same plane as HL. An imaginary line is drawn parallel to the cephalic posterior margin and perpendicular lines are extended anterad to where the posterolateral cephalic curve meets the lateral cephalic margin.

**MI** Mandible index. ML/HL × 100

**ML** Mandible length. The straight-line length of mandible at full closure, measured in the same plane as HL, from mandibular apex to anterior clypeal margin.

**MsL** Mesosoma length. Maximum length of mesosoma, measured in lateral view, a diagonal line from the cervical shield to the posterolateral propodeal edge.

**PrW** Pronotum width. Maximum width of pronotum in dorsal view.

**PtH** Petiole height. Direct linear distance from the apex of petiolar needle to ventral subpetiolar process measured in the same plane as PtL

**PtL** Petiole length. Maximum length of petiole in lateral view.

**PtW** Petiole width. Maximum width of petiole in dorsal view.

**SI** Scape index. SL/HW × 100.

**SL** Scape length. Maximum chord length of antennal scape in dorsal view excluding basal constriction.

### Museum abbreviations

The collection abbreviation is taken from Evenhuis (2020). The specimens used in this study are deposited at the following institutions:

**DZUP** Department of Zoology, Universidade Federal do Paraná, Curitiba, Brazil

**MCZ** Havard Museum of Comparative Zoology, Cambrigde, Massachusetts, USA

**MEPN** Museo de Colecciones Biológicas Gustavo Orcés, Escuela Politécnica Nacional, Quito, Ecuador

**RBINS** Royal Belgium Institute of Natural Sciences, Brussels, Belgium

**QCAZ** Zoology Museum at the Pontifical Catholic University of Ecuador, Quito, Ecuador

### Molecular analyses

We sequenced the classical mitochondrial barcode region for animals, a 658-base pair (bp) region of the *cytochrome oxidase subunit I* gene (COI), for two *O. davidsoni* sp. nov. specimens and six specimens of six additional *Odontomachus* species (*O. erythrocephalus* Emery, 1840, *O. mormo*, *O. chelifer* (Latreille, 1802), *O. meinerti* Forel, 1805, O. cf. mayi Mann, 1912, *O. hastatus* (Fabricius, 1804); see Suppl. material 4 for collection data). Specimens were identified by PH. We used whole specimens for non-destructive DNA extraction using the Qiagen 96 DNeasy Blood & Tissue Kit (Quiagen, Venlo, Netherlands) following the standard protocol with one exception: we shortened the protein lysis step to 2h–3h to avoid any damaging of the specimens, which keeps specimens intact and in good condition so as to serve as morphological vouchers (e.g., von Beeren et al. 2016). We amplified COI using the primer combination LCO1490 / HCO2198 (Folmer et al. 1994). PCRs were set up as described previously by von Beeren et al. (2016). Purification and sequencing of PCR products in forward and reverse direction were outsourced to Macrogen Europe B.V. (Amsterdam, Netherlands). The laboratory information management system Geneious Prime 2019.1.3 was used to process and analyse sequences (https://www.geneious.com).

To evaluate whether COI can be used as reliable species discriminator for *O. davidsoni* sp. nov. we further included available data of published COI sequences of Neotropical *Odontomachus* species. For this, we analyzed *Odontomachus*COI sequences from the New World. We downloaded 264 published COI sequences by using the following search terms in the Barcode of Life database system (www.boldsystems.org; search criteria: Odontomachus; “United States”; USA; Mexico; Cuba; Haiti; “Dominican Republic”; “Puerto Rico”; “British Virgin Islands”; Montserrat; “Antigua and Barbuda”; Dominica; “St Lucia”; Barbados; Grenada; “Trinidad and Tobago”; Guatemala; Honduras; Belize; “El Salvador”; Nicaragua; “Costa Rica”; Panama; Colombia; Venezuela; Ecuador; Guyana; Suriname; “French Guiana”; Brazil; Peru; Bolivia; Paraguay; Chile; Argentina; Uruguay”; accessed on 15 May 2019).

Published and newly acquired data from this study were then analyzed together. We first performed several quality checks. Sequences lacking species identifications, sequences containing ambiguous bases, and sequences smaller than 400bp were sorted out. We then used the MUSCLE algorithm (Edgar 2004) to align COI sequences and, on this basis, sorted out additional sequences which showed gaps and/or additional bases in the sequence alignment. No apparent stop codons were detected in the analyzed *Odontomachus* dataset. Finally, we extracted unique sequences (or COI haplotypes) from the dataset resulting in 94 distinct COI sequences of 16 *Odontomachus* species. For eight of those specimens lateral habitus images were uploaded to BOLD. BOLD process IDs of sequences are given in Suppl. material 5.

We used a Neighbor-Joining (NJ) tree as simple clustering approach of DNA barcode data to depict genetic differences and to examine the reliability of COI as possible molecular identifier of *O. davidsoni* sp. nov. Note that it was not our goal to evaluate whether COI can serve as a reliable identification character in the entire genus. We thus did not define species boundaries in other *Odontomachus* species based on intraspecific p-distance thresholds and barcoding gaps as it is often done so in other barcoding studies. Except for our own identifications (see above) we used and relied on species identifications that were deposited together with COI sequences in GenBank. The NJ tree was analyzed in MEGA 10.0.5 (Kumar et al. 2018) based on p-distances with pairwise deletion of missing data. P-distances simply give the proportion of bases that differ in pairwise sequence comparisons. Metadata of the NJ tree are given in Suppl. material 6 as Newick formatted file.

## Results

### Key to Neotropical *Odontomachus* species

This key is a modification of the keys of Rodriguez (2008) and Brown (1976), both of which contain many additional helpful figures as well as detailed morphological descriptions. Please note that this key does not include the species native to the USA such as the Florida endemic *O. relictus* Deyrup & Cover, 2004, for which we recommend using the key of MacGown et al. (2014).

From the Rodriguez (2008) key only the herewith described *O. davidsoni* sp. nov. is added as new. However, the classification of *Odontomachus brunneus* has been cleared up. Originally, it was thought to be distributed in South and Central America, but it is now clear that it is restricted to the Southern USA, and previous material that has been identified as such probably belongs to *O. ruginodis* Smith, 1937 (MacGown et al. 2014).

For easy identification, we recommend to point mount workers of *Odontomachus* by bending the tip of the point and gluing it to the pleura so as to leave the space between the hind coxae exposed. This is because the metasternal process is an important identification characteristic and it might be obscured otherwise.

### English key

**Table:** 

1	Petiole pedunculate to subpedunculate, in lateral view the anterodorsal margin of the gaster forms a single convexity that ascends posterad at approximately 45° (Fig. 1A)	**2**
–	Petiole sessile, not subpendunculate, in lateral view the anterodorsal margin of the first gaster segment forming a much steeper slope (>45°) with a more or less distinct vertical anterior face (Fig. 1B)	**4**
2	Dorsal surface of the head with deep striation that reaches to the nuchal collar; color uniform ferruginous to dark red	***O. davidsoni* sp. nov.**
–	Posterior third to half of dorsal surface of head smooth and shining; color variable	**3**
3	Without metasternal process; mesonotum almost hairless; larger body size (HL > 4 mm). Ground living	***O. mormo***
–	With metasternal process; dorsal surface of mesonotum covered in small erect setae; smaller body size (HL < 3.8 mm). Arboreal	***O. hastatus***
4	Dorsal surface of head distinctly striate to or nearly to the nuchal carina	**5**
–	Posterior third to half of dorsal surface of head smooth and shining, or nearly so	**22**
5	Disc (dorsal surface) of first gastric segment predominantly smooth, punctulate, alutaceous, or reticulate; striation absent, or if present, mixed with other sculpture and distinct only on the posterior half of the disc	**6**
–	Disc of first gastric segment distinctly and evenly striate over its entire surface, at least as seen from dorsal view	**17**
6	Mesonotum longitudinally striate	***O. yucatecus* Brown, 1976**
–	Mesonotum prevailingly transversely striate	**8**
7	Head more or less bright red (frontal area often infuscate), contrasting with blackish-brown body and yellow legs; size medium	***O. erythrocephalus***
–	Color combination otherwise; if head is distinctly red, then trunk is red also, or legs are dark	**8**
8	Sternum immediately in front of and between metathoracic coxae produced as a slender, acute pair of teeth or spines; disc of first gastric segment densely and finely shagreened and pubescent, usually opaque; body brown, legs yellow to brown	***O. haematodus* (Linnaeus, 1758)**
–	Sternum in front of metathoracic coxae with a low transverse ridge, sometimes notched in the middle or bilobed, but not produced as acute, paired teeth	**9**
9	Petiole predominantly smooth and shiny, with the anterior border erected or slightly convex, and the apex produced into a large spine with posterior orientation; head, mesosoma and petiole with a clear red coloration and the gaster dark brown	***O. insularis* Guérin-Méneville, 1844**
–	Petiole differently shapes, color combination varies	**10**
10	Anterior face of petiolar node as seen from the side rising steeply from anterior margin, then passing through an obtuse angle into a long section concave in outline to the root of the apical spine; labial palpi 4-merous	***O. bradleyi* Brown, 1976**
–	Petiole differently shaped	**11**
11	Metasternal process like an arc with or without middle division; petiole smooth or a little striate, with both the anterior and posterior margin convex; the petiolar spine forms gradually, without clear distinction from petiole	**O. cf. brunneus Patton**, **1894** (possibly *Odontomachus ruginodis*; *O. brunneus* is apparently restricted to southeastern US. The status of the Central and South American populations comparable with *O. brunneus* need to be established.)
–	Metasternal process absent, bilobed or triangular; petiole differently shapes or if both sides convex, with a clear differentiation of the spine from the rest of the petiole	**12**
12	Metasternal process absent	**13**
–	Metasternal process bilobed or triangular	**14**
13	Black coloration; Node of petiole with a pair of prominent posterolateral tumosities at about mid-height and without striation; apex as seen from side abruptly narrowed to an axially erect, acute tooth	***O. biumbonatus* Brown, 1976**
–	Node of petiole without paired posterolateral tumosities; Posterior face of petiole less concave, with short petiolar spine (0.1 mm)	***O. clarus* Roger, 1861**
14	Anterior margin of petiole at least weakly convex	**15**
–	Anterior margin of petiole basal of the node concave or straight	**16**
15	Metasternal process completely bilobed; color generally dark	***O. bauri* Emery, 1892**
–	Metasternal process formed by an obtuse wide lobe followed by a transverse flange which is produced into a triangular process; usually light color	***O. biolleyi* Forel, 1809**
16	Petiole strongly transversely striate, with a clearly differentiated spine; small species (TL 8.6–9.35 mm)	***O. ruginodis* Smith, 1937**
–	Petiole without or only weak striation and with spine not clearly differentiated from the petiole; large species (TL 12 mm)	***O. laticeps* Roger, 1861**
17	First gaster segment with only one type of sculpture, which is either punctulate or striate	**18**
–	First gaster segment with a combination of punctulate and reticulate sculpture	**21**
18	First gaster segment punctulate on its entire surface	***O. opaciventris* Forel, 1899**
–	First gaster segment striate on most of its surface, at least in dorsal view	**19**
19	Transverse striation patterns on the gaster; large and slender	***O. chelifer* (Latreille, 1802)**
–	Longitudinal striation on the dorsal gaster surface	**20**
20	Mesonotum strongly convex, but broadly sulcate and longitudinally striate on at least the anterior half near midline	***O. caelatus* Brown, 1976**
–	Mesonotum gently but evenly convex, transversely striate	***O. laticeps***
21	Mesonotum with longitudinal striation	***O. scalptus* Brown, 1978**
–	Mesonotum with transverse striation	***O. meinerti* Forel, 1805**
22	Ocular prominences each produced anterolaterally into a stout, acute, oblique, toothlike process	***O. cornutus* Stitz, 1933**
–	Ocular prominences bluntly rounded, as usual	**24**
23	Antennal scapes very short, not reaching posterior border of head in full-face view; very small species with broad head	***O. spissus* Kempf, 1962**
–	Antennal scapes surpassing posterior border of head viewed full-face	**24**
24	Apex of mandible with only 2 large teeth (intercalary tooth lacking)	***O. allolabis* Kempf, 1974**
–	Apex of mandible with 3 teeth	**25**
25	Mesepisternum with a prominent, narrowly rounded anteroventral lobe projecting conspicuously on each side when trunk is viewed from above	***O. mayi* Mann, 1912**
–	Mesepisternum with at most a low, inconspicuous convexity on its anteroventral margin	**26**
26	Petiole clearly differentiated spine; larger species (HL > 2.8 mm)	***O. affinis* Guérin-Méneville, 1844**
–	Both faces of petiole converge into a thick spine that is flattened laterally; smaller species (HL < 2.8 mm)	***O. panamensis* Forel, 1899**

### Spanish key

**Table:** 

1	Pecíolo en vista lateral pedunculado a semi-pedunculado, el perfil anterodorsal del primer segmento del gáster forma una convexidad contínua con una pendiente de aproximadamente 45° (Fig. 1A)	**2**
–	Pecíolo sésil, no semi-pedunculado, el perfil anterior del primer segmento del gáster relativamente vertical y bien diferenciado del perfil dorsal, con una pendiente mayor de 45° (Fig. 1B)	**4**
2	Superficie dorsal de la cabeza con estrías que llegan hasta la carena nucal, color ferruginoso	***O. davidsoni* sp. nov.**
–	Superficie dorsal de la cabeza con estrías que tan sólo ocupan de la mitad a dos tercios de la región anterior de la cabeza, color es variable	**3**
3	Espacio entre las coxas posteriores liso, sin proceso ni estrías; mesonotum casi sin pelos; gran tamaño (LC > 4 mm). Hormiga del suelo	***O. mormo***
–	Espacio entre las coxas posteriores con un proceso bilobulado y siempre estriado; mesonotum con muchos pelos; más pequeña (LC < 3.8 mm). Hormiga arbórea	***O. hastatus***
4	Superficie dorsal de la cabeza con estrías que llegan hasta la carena nucal o muy cerca de ésta	**5**
–	Superficie dorsal de la cabeza con estrías que tan sólo ocupan de la mitad a dos tercios de la región anterior de la cabeza	**22**
5	Primer segmento del gaster predominantemente liso y brillante, opaco o suavemente reticulado	**6**
–	Primer segmento del gaster con escultura que puede ser de un sólo tipo o una mezcla de varios (estríado, punteado, estríado- punteado)	**17**
6	Mesonoto estríado longitudinalmente	***O. yucatecus***
–	Mesonoto estríado transversalmente	**7**
7	Cabeza de color rojo claro que contrasta con cuerpo marrón oscuro a negro y extremidades amarillas	***O. erythrocephalus***
–	Diferente combinación de color; si la cabeza es rojo claro, entonces el mesosoma debe ser también rojo o las extremidades de un color oscuro; o la cara anterior del pecíolo es recta o cóncava	**8**
8	Metaesterno, exactamente entre las coxas posteriores posee un par de espinas o dientes agudos; primer segmento del gaster reticulado, usualmente opaco; cuerpo marrón, extremidades de color amarillo a marrón	***O. haematodus***
–	Metasterno sin o con proceso, el cual puede ser bilobulado, dividido en la mitad o redondeado	**9**
9	Pecíolo predominantemente suave y brillante, con borde anterior recto o ligeramente convexo, el ápice de éste se estrecha formando una espina larga, delgada que está dirigida posteriormente; cabeza, mesosoma y pecíolo de color rojo claro y gaster marrón oscuro	***O. insularis***
–	Pecíolo de diferente forma; combinación de color variada	**10**
10	Cara anterior del nodo peciolar se levanta casi verticalmente desde el margen anterior, luego pasa por un ángulo obtuso a una sección larga y cóncava que forma una espina apical	***O. bradleyi***
–	Pecíolo con forma diferente	**11**
11	Proceso metasternal como un arco con o sin división en el medio; pecíolo suavemente o poco estríado, la cara anterior es convexa, al igual que la posterior; la espina del pecíolo se va formando gradualmente, lo cual hace que no sea claramente diferenciada de éste	**O. cf. brunneus** (Posiblemente se trata de *O. ruginodis*. *O. brunneus* aparentemente esta restringida al sureste de los EEUU. El estatus de las poblaciones centro y suramericanas que son comparables con *O. brunneus* aún esta por definirse.)
–	Proceso metasternal ausente, bilobulado o triangular; pecíolo con diferente forma o si ambos lados son convexos hay una espina claramente diferenciada del resto del pecíolo	**12**
12	Proceso metasternal ausente	**13**
–	Proceso metasternal bilobulado o triangular	**14**
13	Color negro; nodo del pecíolo con un par de prominencias posterolaterales y sin estrías; ápice en vista lateral se estrecha hasta formar un diente agudo axialmente erguido	***O. biumbonatus***
–	Color claro; nodo del pecíolo sin prominencias o si las posee tiene estrías; cara posterior del pecíolo al menos débilmente cóncava, la espina peciolar es corta (0.1mm)	***O. clarus***
14	Cara anterior del pecíolo al menos débilmente convexa	**15**
–	Parte anterior basal del nodo peciolar cóncava o recta	**16**
15	Proceso metasternal completamente bilobado; color oscuro generalmente	***O. bauri***
–	Proceso metasternal formado por un lóbulo ancho obtuso, seguido de un reborde transverso que se observa como un proceso de forma triangular; color claro generalmente	***O. biolleyi***
16	Pecíolo fuertemente estríado transver- salmente, presenta una espina claramente diferenciada, especies pequeñas (8.6- 9.35mm)	***O. ruginodis***
–	Pecíolo sin estrías o suavemente estríado, la espina no está claramente diferenciada del pecíolo, especies grandes (TL 12 mm)	***O. laticeps***
17	Primer segmento del gaster con un sólo tipo de escultura, que puede ser punteada o estriada	**18**
–	Primer segmento del gaster con una combinación de escultura punteada y reticulada	**21**
18	Primer segmento del gaster punteado a lo largo de toda su superficie	***O. opaciventris***
–	Primer segmento del gaster estríado a lo largo de toda su superficie, al menos en vista dorsal	**19**
19	Estrías transversales curvas en el gaster; especies grandes y delgadas	***O. chelifer***
–	Estrías longitudinales en el dorso del gaster	**20**
20	Mesonoto fuertemente convexo, pero fuertemente surcado y estríado longitudinalmente al menos en la mitad del área de la parte	***O. caelatus***
–	Mesonoto suave pero uniformemente convexo, estríado transversalmente	***O. laticeps***
21	Mesonoto estríado longitudinalmente	***O. scalptus***
–	Mesonoto estríado transversalmente	***O. meinerti***
22	Prominencias oculares con un proceso agudo, oblicuo a manera de diente	***O. cornutus***
–	Prominencias oculares redondeadas	**23**
23	Escapos antenales muy cortos que no alcanzan el borde posterior de la cabeza	***O. spissus***
–	Escapos antenales sobrepasan el borde posterior de la cabeza	**24**
24	Dos dientes grandes en el ápice de la mandíbula	***O. allolabis***
–	Tres dientes en el ápice de las mandíbulas	**25**
25	Mesepisterno con un lóbulo anteroventral redondeado y prominente que se proyecta a los lados del mesosoma en vista dorsal	***O. mayi***
–	Mesopleura con una convexidad inconspicua en su margen anteroventral	**26**
26	Pecíolo convexo en ambas caras, con una espina delgada claramente diferenciada; más grande (LC>2.8 mm)	***O. affinis***
–	Pecíolo con ambas caras convexas, las cuales convergen en una espina gruesa y aplanada lateralmente; más pequena (LC<2.8 mm)	***O. panamensis***

**Figure 1.**
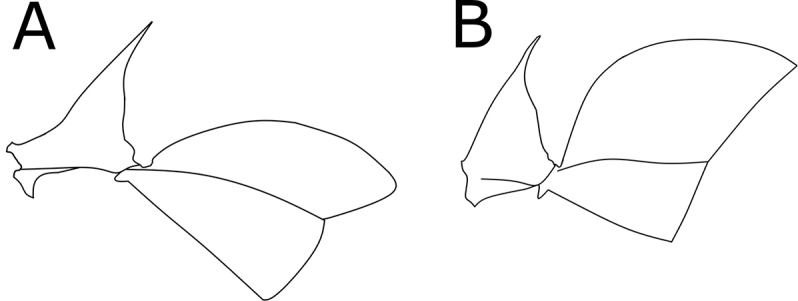
Lateral view of petiole and first gaster segment of *O. hastatus* (left) and *O. bauri* (right).

#### Odontomachus davidsoni

Taxon classificationAnimaliaHymenopteraFormicidae

Hoenle, Lattke & Donoso sp. nov.

268A14A5-E98A-5D55-A374-1954A79D3BEA

http://zoobank.org/6FF7413B-42DA-4386-87D4-09C8FB06F70D

Figures 2 , 3

##### Type material examined.

Complete list of localities in Suppl. material 9. Unique museum specimen identifiers are given in brackets after each specimen identification code.

##### Type locality.

Ecuador • Esmeraldas, Reserva Río Canandé; 0.5281N, 79.2070W; ca. 330 m; 21 February 2019; P. Hoenle & G. Villagomez leg.; collection code PE39; single worker near large fig tree in mature forest.

**Figure 2.**
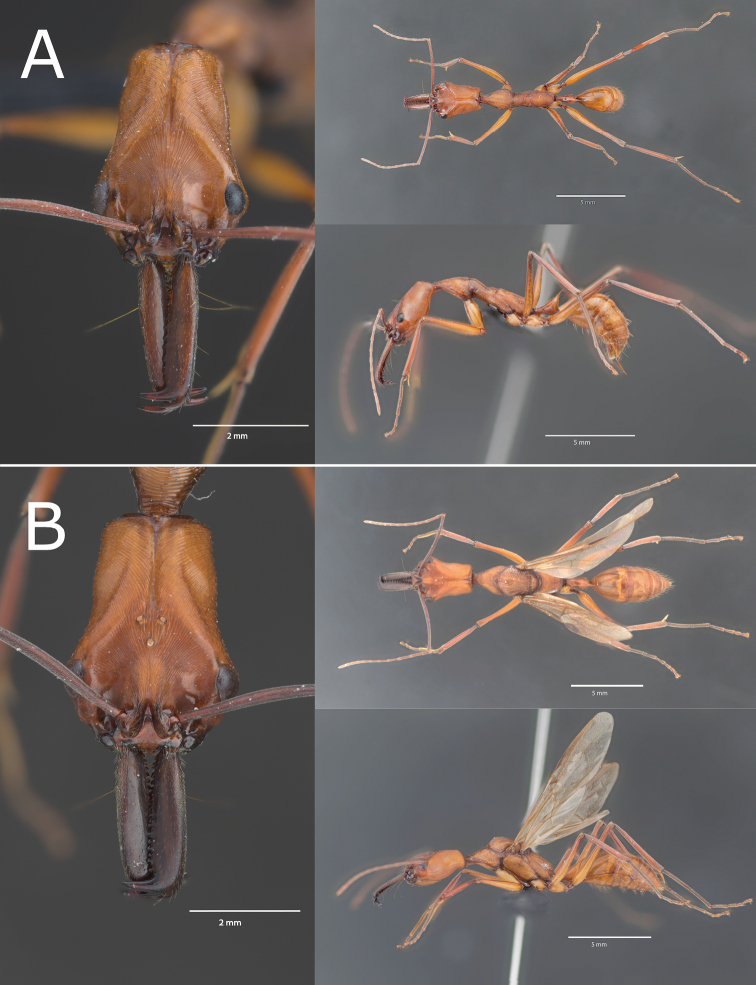
*Odontomachus davidsoni* sp. nov. stacking pictures **A** Worker paratype (specimen PE23_01) **B** Queen paratype (specimen PE24_01). Additional pictures in Suppl. material 7. Scale bars: 2 mm (left); 5 mm (right).

**Figure 3.**
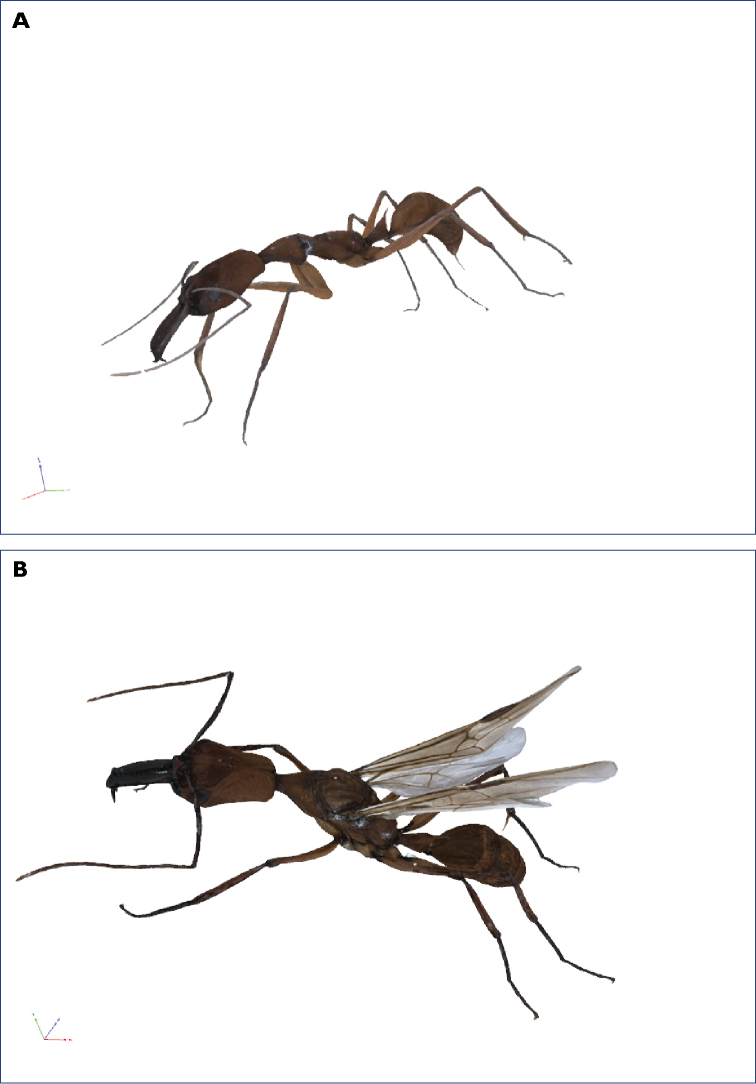
3D models of *Odontomachus davidsoni* sp. nov. **A** 3D scan of *Odontomachus* holotype (PE39_01). **B** 3D scan of queen (PE24_01). Additional 3D models of two more paratypes in Suppl. material 8.

##### Holotype specimen.

Ecuador • 1 worker; Esmeraldas, Reserva Río Canandé; 0.5281N, 79.2070W; ca. 330 m; 21 February 2019; P. Hoenle & G. Villagomez leg.; collection code PE39; single worker near large fig tree in mature forest; specimen code PE39_01; [MEPN5074].

##### Paratype workers.

Ecuador • 3 workers; Esmeraldas, Reserva Rio Canande; 0.5252N, 79.2079W; ca. 320 m; 04 February 2019; P. Hoenle & A. Argoti leg.; collection code PE23; hand sampling on Cecropia tree, same location as Odonto_Phil; specimen codes PE23_01, PE23_02, PE,_23_03; [MCZ-ENT00731935].

Ecuador • 2 workers; Esmeraldas, Reserva Río Canandé; 00.5263N, 79.2117W; ca. 310 m; 06 February 2019; P. Hoenle leg.; collection code PE25; workers on recently large, fallen tree; specimen codes PE25_01, PE25_02; [RBINS IG 34167].

Ecuador • 4 workers; Esmeraldas, Reserva Río Canandé; 0.5238N, 79.2130W; ca. 330 m; 11 February 2019; P. Hoenle leg.; collection code PE36; nest in fallen branch in secondary forest (former cacao plantation); specimen codes PE36_01[RBINS IG 34167]; PE36_02, PE36_03, PE36_04; [MEPN_5075].

Ecuador • 4 workers; Esmeraldas, Reserva Río Canandé; 0.5252N, 79.2079W; ca. 320 m; 29 May 2018; P. Hoenle & A. Argoti leg.; collection code Odonto_Phil; hand sampling on Cecropia tree. Same colony as PE23; One point-mounted worker ;specimen code: Odon_Phil_02 [DZUP 548819], (BOLD ID: ODECU002-19) and one point-mounted worker with the same locality data but collected on 20 May 2018, specimen code Odon_Phil_01 [DZUP 548820]; (BOLD ID: ODECU001-19); Two workers mounted with permanent glue on top of needle specimen codes Odon_Phil_3, Odon_Phil_4; [PH private collection]

##### Paratype queens.

Ecuador • 2 queens; Esmeraldas, Reserva Río Canandé; 0.5263N, 79.2129W; ca. 340 m; 25 January 2019; P. Hoenle leg.; collection code PE24; 2 alate queens, ex. light trap at the Río Canandé station, 8 pm.; specimen codes PE24_01 [MCZ-ENT00731935], PE24_02 [MEPN_5076].

Ecuador • 1 queen; Esmeraldas, Reserva Río Canandé; 0.5263N, 79.2129W; ca. 340 m; 13 April 2019; P. Hoenle leg.; collection code PE87, 1 alate queen, ex. light trap at the Río Canandé station, 9 pm.; specimen code PE87_01; [RBINS IG 34167].

Ecuador • 1 queen; Esmeraldas, Reserva Río Canandé; 0.5263N, 79.2129W; ca. 340 m; 09 May 2018; P. Hoenle & A. Argoti leg.; collection code Odon_Phil_queen; ex. light trap at Canandé Lodge; specimen code Odon_Phil_queen_01; [PH private collection].

Ecuador • 1 queen; Esmeraldas, Kumanii Lodge near Cotocachi-Cayapas Reserve; 0.7539N, -78.9208W; ca. 40 m; 14 April 2006; L. Camacho leg.; ex. light tap; [QCAZI 15167].

##### Specimens used for 3D scan:

1 holotype worker (PE39_01), 1 paratype worker (PE23_01), 1 paratype worker (PE36_01), 1 paratype queen (PE24_01)

##### Specimens used for DNA barcoding:

Paratype workers DZUP 548819 (BOLD ID: ODECU002-19) & DZUP 548820 (BOLD ID: ODECU001-19)

##### Diagnosis of workers.

***Measurements*** (*N* = 3): HL 3.91–4.09, HoW 2.67–2.76, HvW 1.65–1.74, ML 2.62–2.70, SL 4.22–4.43, EL 0.62–0.71, MsL 6.00–6.20, PrW 1.49–1.57, PtW 0.59–0.64, PtL 1.53–1.57, PtH 2.20–2.23, FL 5.28–5.37, CI 67.48–68.53, SI 158.05–160.74, MI 65.28–69.05.

Long (TL > 17 mm), but slender, ferruginous to yellow brown body with striae on cephalic dorsum from antennal insertions to vertex, mandible with over 15 pre-apical teeth and denticles, pronotal dorsum with concentric to transverse striae. Petiole strongly pedunculate with posteriorly inclined apical spine, gaster smooth and shining.

##### Description of the holotype worker.

Head elongate in dorsal view, anterior and posterior margins approximately of same width, posterior cephalic margin mostly transverse; head widest across eyes, at anterior one-third of head length; lateral cephalic margin posterior to eye sinuous. Median furrow deep, extends anterad to antennal fossa where it fades; occipital ridge distinctly delineated by antennal fossa, extending posteromedially, joining broad ridge that runs parallel to median furrow. Extraocular furrow broad and shallow, temporal prominence broad and weakly elevated. Cephalic surface with well-defined striae that diverge posterad from between frontal carina, reaching vertex, striae fade away on most of lateral cephalic surface with some striae reaching posteroventral cephalic surface. Ocular ridge smooth closest to eye and striate towards cephalic median region. Cephalic dorsal surface anterad of eye and between eye and antennal sclerite mostly smooth. Scape slender and slightly arched, SL longer than HL, scape widest just anterad of mid length, finely punctulate; funicular segment elongated and slender, segment I half as long as segment II.

Median clypeus mostly smooth and shining, posteriorly projecting as flattened triangular surface between frontal carina; carina defines narrow elevated region that descends posteriorly and extends to antennal fossa; frontal carina narrow, width not greater than scape width; carina steeply elevated over posteromedian clypeal surface. Ventral cephalic surface mostly smooth and shining. Labium drop-shaped, anteroventral surface very convex, PF 4,4. Buccal cavity with lateral hypostomal tooth. Mandibular masticatory margin with basal row of six denticles and eleven blunt triangular, relatively short teeth apicad of denticles. One or more teeth closest to apex may be broken. Mandibular apex tridentate, ventral tooth with basal tooth. Mandibular dorsal surface mostly smooth, with sparse piligerous punctulae, but dorsolaterally with abundant punctulae, ventral surface smooth and shining.

Pronotal dorsum with concentric striation that become progressively transverse and elongate medially towards posterior margin, in lateral view striae appear anteriorly transverse, medially curving and U-shaped, posteriorly oblique to almost vertical. Posterolateral pronotal margin with short convex lobe. Mesosoma relatively slender and elongate, in lateral view pronotal dorsal margin straight to weakly convex, forming a posteriorly ascending slope, mesonotum anterior margin slightly higher than posterior pronotal margin, mesonotal dorsal margin mostly straight to weakly convex, descending to metanotal groove. Dorsal mesosomal margin between metanotal sulcus and metanotal spiracle forms brief convexity, propodeal anterodorsal margin brief and convex, dorsal margin mostly straight, three times longer than declivity, declivity forms blunt obtuse angle with dorsal margin. Propleuron mostly smooth and shining with narrow transverse band of sparse weak rugulae anteriorly and posteriorly.

Mesonotum with transverse striae that extend uninterrupted laterally to anepisternum and ventrally to mesosternum, katepisternum mostly smooth and shining except for sparse striae anteriorly and posteroventrally. Bulla of metathoracic spiracle semispherical, weakly sculpted, opening shaped as transverse slit. Propodeum and metanotum transversely striate. Mesometapleural suture distinct, propodeal- metapleural suture weakly impressed. No carina or visible suture between mesopleuron and mesosternum, mesosoma in hypothetical cross-section at mid-length forms relatively uniform ovoid. Mesosternum with median longitudinal region raised as low and broad convex ridge; metasternal process bidentate, teeth short and blunt. Propodeal spiracle slit-shaped, transverse to oblique, not elevated.

Petiole in lateral view slightly pedunculate, node shaped as posteriorly sloping cone with acute apical needle, anterior node margin weakly convex, posterior margin vertical, straight to weakly convex; anteroventral process prominent, triangular; node smooth and shining. Abdominal tergite 3 in lateral view with anterodorsal margin forming single convexity to posterior margin, ascending posterad at approximately 45°; ventral margin of tergite 3 briefly concave at prora, then broadly convex and mostly at the same level as prora. Constriction between abdominal segments 3, 4 weak to negligible; gaster smooth and shining.

Coxae mostly smooth with abundant minute piligerous punctulae, punctulae denser on tibiae. Protibial apex with single seta, spur with basal translucent lamella. Probasitarsus with row of short, stiff hairs and parallel row of short setae opposite spur. Meso and metatibial apex each with two spurs, one pectinate, one simple; each also with 3 setae, each seta widely separated from each other. Body pilosity generally short and scattered with little pubescence; dorsal surface with few standing hairs: one on head where antennal fossa and nuchal carina almost meet, few on gastral sterna. Head and mandibular dorsum with sparse appressed pubescence, hairs straight on mandible and arched on head. Mandibular ventral surface next to masticatory margin with row of five flagellate long hairs plus two long trigger hairs at base. Scape with dense appressed pubescence, no standing hairs. Mesosoma with sparse appressed to subdecumbent small hairs, node with longer hairs; gaster mostly with sparse short, appressed to decumbent hairs with suberect hairs towards posterior end of gaster. Mandible and other buccal appendages, antenna, tibiae, and tarsi ferruginous brown to brown. Body mostly ferruginous to brownish yellow, head dark anterad and gaster darker posterad; trochanters and apex of femora tend to be darker.

##### Queen.

***Measurements*** (*N* = 1): HL 4.30, HoW 3.8, HvW 1.99, ML 2.87, SL 4.46, EL 0.75, MsL 6.78, PrW 2.22, PtW 0.79, PtL 1.68, PtH 2.40, FL 5.46, CI 71.63, SI 144.81, MI 66.74.

Mesosoma developed for wings, head with three ocelli. Queen with larger dimensions than worker: HoW > 3.3; MsL > 6.5; PrW >1.9 mm., otherwise similar.

**Male.** Unknown.

***Etymology.*** The species epithet is a patronym in genitive case honoring Stuart Carleton Davidson, the founder of Clyde’s Restaurant Group, Washington, DC. Stuart had a lifelong interest in our environment, and would have loved this amazing ant.

##### Comparison to similar species.

**Figure 4.**
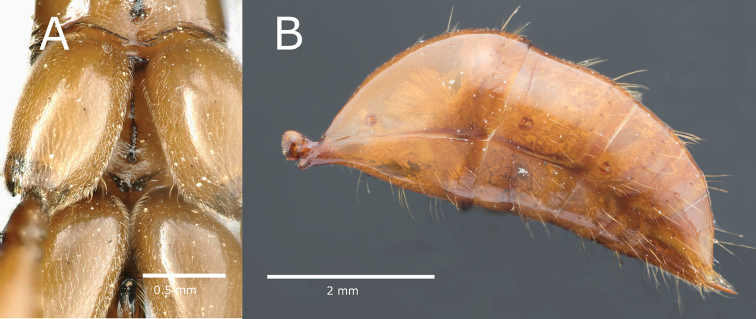
*Odontomachus davidsoni* ventral picture of metasternal process (specimen: Odon_Phil_3) and lateral image of gaster (specimen Odon_Phil_4).

*Odontomachus davidsoni* most closely resembles *O. hastatus* by sharing a relatively large size, a red to brown color, a head which has in dorsal view a great difference between ocular and vertexal width, a relatively slender habitus, a bilobed metasternal process (Fig. 4A), and a pedunculate petiole with a posterior inclining node topped by a long dorsal needle. Together with *O. mormo* both species also share an evenly convex anterior margin of abdominal tergite III that in lateral view ascends posterad at an approximate 45° angle (Fig. 4B).

Compared to *O. hastatus*, *O. davidsoni* is clearly larger: The HL range of *O. hastatus* is 2.81–3.67 mm (Brown 1976) versus 3.91–4.09 mm. Further, it has coarse striae throughout the dorsal cephalic surface, whereas the striae on *O. hastatus* are fine and limited to the area between the frontal carina and the antennal fossa, not extending to the occipital ridge. The pronotum in *O. hastatus* frequently presents smooth and shining areas on the pronotal disc or its sides, where it is always striate in *O. davidsoni*.

While the body size of *O. davidsoni* is similar to *O. mormo* (HL 4.14–4.36 (Brown 1976), both species can be clearly distinguished by the striation pattern on the head: The striation in *O. mormo* does not reach the occipital ridge and there is instead a large and shiny area on the cephalic dorsum, while in *O. davidsoni* the entire cephalic dorsum is covered in coarse striae. Further, *O. mormo* does not possess a metasternal process, while *O. davidsoni* has a rounded bilobed metasternal process. *O. mormo* is almost hairless on the entire dorsal body surface including the petiole, whereas *O. davidsoni* body possesses a few appressed to decumbent hairs on the mesonotum, and many long erect hairs on the petiole. Overall, *O. mormo* has a more brownish coloration (very similar to *O. chelifer*), in contrast to the red coloration in *O. davidsoni*.

When using the key to Neotropical *Odontomachus* species by Rodriguez (2008) this species will be taken easily to couplet 13, whereupon it will not fit any of the two alternatives: *O. ruginodis* nor *O. laticeps*. It is clearly larger than *O. ruginodis*, which also differs in its dark brown color, sessile petiole, and a very short stubby petiolar needle. *O. laticeps* is smaller, dark brown, with a sessile and relatively erect petiole bearing a shorter dorsal needle. Using the identification key in Brown (1976: 111), or the key in Antwiki (2015), this species is easily taken to couplet 14 where it becomes stuck as it fits neither alternative, *O. bauri* nor *O. laticeps*. Both of these ants are much smaller, dark brown, have a sessile, erect petiolar node with a relatively shorter dorsal needle, and the anterior dorsal margin of abdominal tergite III in lateral view is mostly vertical.

##### Molecular analyses.

**Figure 5.**
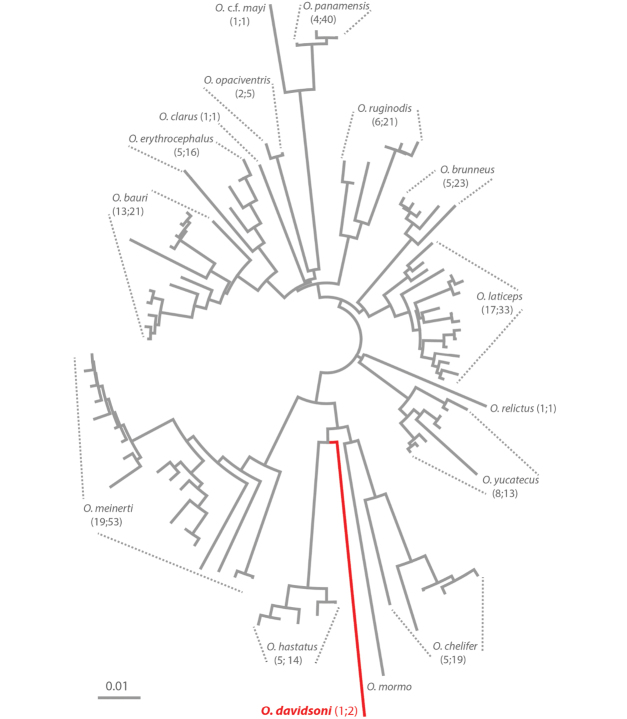
Neighbor-Joining (NJ) tree of *Odontomachus*COI sequences. The NJ tree is based on p-distances (scale bar). First digit in parentheses gives the number of identified COI haplotypes of a given species and the second one the total number of available COI sequences for this species. GenBank accession numbers are given in Suppl. material 5 and Newick tree file are given in Suppl. material 6.

We successfully amplified DNA barcodes of two *Odontomachus davidsoni* workers, a 569 bp fragment and a 668 bp fragment (GenBank accession numbers MN454765 and MN454766, respectively). The two specimens came from the same nest and had identical sequences. *Odontomachus davidsoni* barcodes were clearly distinguishable from COI sequences of other *Odontomachus* species (Fig. 5) as indicated by the minimum interspecific p-distance of 0.09 in pairwise comparisons (range of p-distances in 94 pairwise comparisons: 0.09–0.14; Fig. 5). A search in the BOLD identification database for the closest sequence match yielded similar results of 90.76% and 90.67% sequence similarity to *O. chelifer* (private, not published yet) and *O. hastatus* (GenBank accession number: KU504889), respectively (accession date 28 May 2019).

##### Biology and distribution.

**Figure 6.**
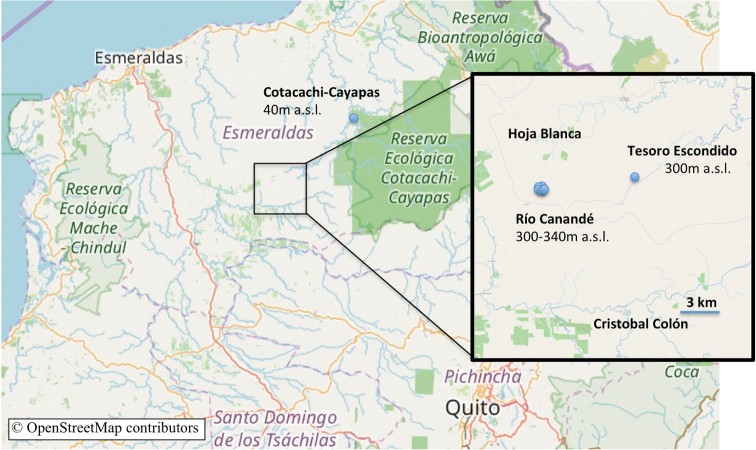
*Odontomachus davidsoni* collection sites. Blue dots show collection sites in Esmeraldas Province (Ecuador) within the reserves Río Canandé, Tesoro Escondido and Cotacachi-Cayapas.

**Figure 7.**
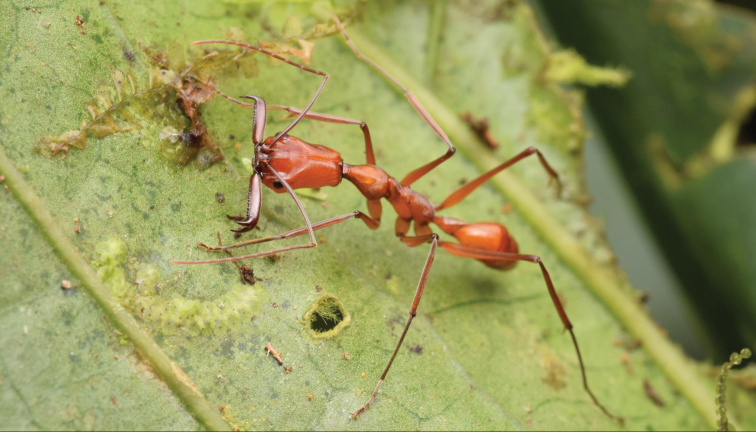
*Odontomachus davidsoni* worker sitting on a leaf in its natural habitat.

Workers of *O. davidsoni* were only found in the Río Canandé Reserve and its neighboring reserve Tesoro Escondido (Fig. 6). Alate queens were collected with light traps in April 2006 (Kumanii Lodge, Cotocachi-Cayapas Reserve, leg. Camacho), April and June 2018, as well as in February, March and April 2019 (Canandé Lodge, Río Canandé Reserve, leg. Hoenle). In 2018 and 2019 we frequently visited a tree in a selectively logged area of the Canandé Reserve where a few workers of the species were spotted. Foraging workers were observed predominantly during nighttime between 8 pm and 11 pm. On at least five occasions during daytime (i.e., between 9 am and 5 pm) the plot was visited, but only once foraging workers were observed. Although their exact nest position was not detected, workers were predominantly foraging on a liana attached to a *Cecropia* tree. The tree had a diameter of 63 cm at breast height and an estimated height of 20 m. Workers could be observed walking straight up on it until they were out of sight in a height of approx. 10 m. Workers were never seen foraging on the ground, and we thus assume that they primarily forage in the canopy. Workers were observed to sit still on the surface of leaves with their mandibles open, probably waiting to ambush prey (Fig. 7).

**Figure 8.**
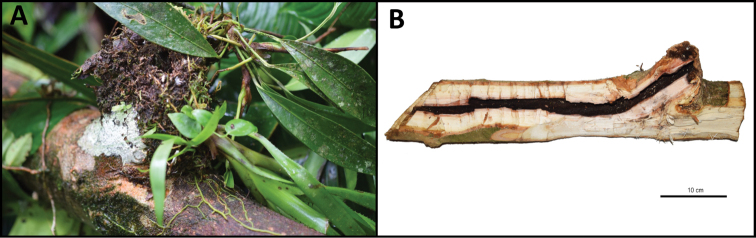
Nest of *Odontomachus davidsoni***A** Nest found under natural conditions with bromeliad covering the nest entrance **B** Nest architecture visible after opening a fallen tree branch with a machete. Scale bar: 100 mm.

**Figure 9.**
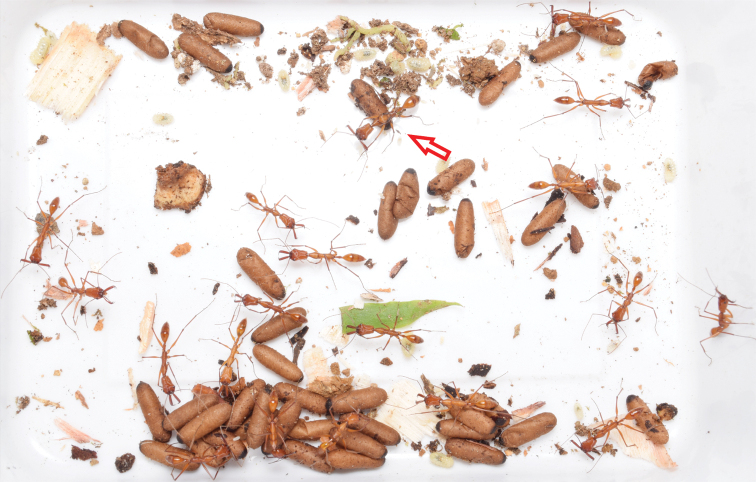
Colony of *O. davidsoni*. This colony was kept in the field laboratory after taking it out from the twig nest (Fig. 8). The sole dealate queen is marked with a red arrow.

On the 11th of February 2019 we collected what looked like a complete nest in a fallen branch (Fig. 8; GPS data: 0.5238N, 79.2130W). The nest contained one dealate queen and 18 workers, as well as brood in all development stages (Fig. 9). We assume that this colony was not fully grown because it contained no alates (despite other colonies having alates at this time). The single nest entry was located under a bromeliad (Fig. 8A). We opened all parts of the nest with a machete, revealing a 40cm long tubular chamber within the center of the branch (Fig. 8B). It does not look like the ants themselves carved it, hence it was probably a pre-existing cavity.

We kept the colony for three months (11 February–15 April 2019) for further observations. The colony accepted various smaller insects as food, including flies, crickets, and termites. However, insects larger than 2 cm (e.g., large cicadas, moths, large crickets) were usually not accepted. Furthermore, the colony had *ad libitum* access to sugar water which was frequently visited. In accordance with field observations, the colony showed most activity during nighttime. No recruitment to offered food resources was observed. Due to the possibly threatened status of this ant species, the colony was released at the end of our observation time on a tree bromeliad nearby the Canandé lodge.

## Discussion

We here formally described the species *Odontomachus davidsoni*. It is morphologically and genetically different from any other of the New World *Odontomachus* species. It rivals in size *O. mormo*, the largest known *Odontomachus* in the Americas, but the dark brown color, more sessile and robust petiole, mostly smooth cephalic dorsum, and lack of a metasternal process in *O. mormo* will easily permit the distinction between the two species. Unfortunately, there are few measurements available for gauging the dimensions of *O. mormo* (Brown 1976: 118; Rodriguez 2008: 156) but it seems safe to affirm that together with *O. chelifer* these species share the position as the largest known *Odontomachus* in the Americas.

Sequence similarities of COI barcodes suggested that *O. davidsoni* might be most closely related to *O. hastatus*, *O. mormo*, and *O. chelifer*. However, more informative phylogenetics/-genomics analyses are necessary to draw robust conclusions. This is because a single mitochondrial locus does not allow us to reliably infer phylogenetic relationships and because our species coverage is incomplete (ten Neotropical *Odontomachus* species do not possess published barcodes). Morphological characters suggest that *Odontomachus davidsoni* is most closely related to *Odontomachus hastatus*, which shares the arboreal and nocturnal foraging lifestyle (Camargo and Oliveira 2011; Rodrigues and Oliveira 2014). The species are easily separated by the lack of striation at the occipital margin in *O. hastatus* and the uniform red coloration of *O. davidsoni*. Both species seem to live in sympatry: our closest record of *O. hastatus* is in a linear distance of approximately 727 m to *O. davidsoni* (390 m vs. 300 m elevation, respectively). Other *Odontomachus* species found in the vicinity are *O. mormo*, *O. chelifer*, *O. bauri*, *O. erythrocephalus*, and *O. meinerti* (PH, pers. obs.), which highlights the high local species richness in the reserve.

Our species description is accompanied by 3D scans of three workers and a queen (Fig. 3, Suppl. material 8). This offers morphological details of the new species to the reader and the ability to take exact trait measurements. 3D imaging techniques, and in particular micro-computed X-ray tomography (µCT), are becoming more frequently used in taxonomy and functional morphology, including studies on ants (Faulwetter et al. 2013; Akkari et al. 2015; Garcia et al. 2017; Sarnat et al. 2017; Staab et al. 2018). Garcia et al. (2017) provided a comprehensive overview of the benefits and caveats of µCT scans for such purposes. The 3D scans used in our study were produced via photogrammetry. This technique has some advantages as well as disadvantages over µCT scans, e.g., it is comparatively inexpensive and requires little manual work, but it provides no internal structure and has a comparatively low spatial resolution (Ströbel et al. 2018). The resulting surface model can thus lack taxonomically important structural features such as the head striation in the case of *O. davidsoni*. On the other hand, the resulting 3D surface models are texturized, thus also providing information on specimen color, which is lacking in µCT scans. A further disadvantage of DISC3D scans is that structures that are obscured in the physical specimen, e.g., through leg positioning or characters laying underneath the glue, are not recovered in a resulting 3D model. Overall, we argue that the 3D scans are a good addition to the traditional morphological description and stacking images by providing valuable 3D models that everyone can easily access and use for reliable trait measurements. Additionally, the 398 EDOF images, that the 3D models are based on, allow for an even more detailed inspection of the specimens in comparison to the texture on the 3D model.

The new species was discovered in the Chocó-Darién bioregion in Ecuador, probably one of the most biodiverse regions on earth, and at the same time one of the most threatened ones (Dinerstein et al. 1995; Olson and Dinerstein 1998; Myers et al. 2000). Research in this region is scarce, with many undescribed endemic species still awaiting scientific discovery and description (Donoso et al. 2009). The fact that a large and conspicuous ant like the herein described trap-jaw ant *O. davidsoni* remained unknown to science until now suggests that a hidden diversity remains to be discovered in this region. Similar to other biodiversity hotspots, increasing deforestation and conversion of forests to agriculture threatens the biodiversity of the Chocó-Darién bioregion, bearing the risk that many endemic and even yet undescribed species become extinct before being detected. With the description of a new, conspicuous trap-jaw ant, probably endemic to the region, we hope to provide additional reasons to protect this threatened biodiversity hotspot.

## Conclusion

*Odontomachus davidsoni* is a noteworthy discovery in a vastly understudied and highly threatened area, the Chocó-Darién region of Ecuador. We sincerely hope that conservation efforts will continue and expand to protect this unique and important biodiversity hotspot.

## Supplementary Material

XML Treatment for Odontomachus davidsoni

## Supplementary materials

Supplementary material 1 Video of *Odontomachus davidsoni* holotype 3D model Data type multimedia Explanation note Rendered video of the 3D scan of the holotype specimen. https://binary.pensoft.net/file/431602This dataset is made available under the Open Database License (http://opendatacommons.org/licenses/odbl/1.0/). The Open Database License (ODbL) is a license agreement intended to allow users to freely share, modify, and use this Dataset while maintaining this same freedom for others, provided that the original source and author(s) are credited.Philipp O. Hoenle, John E. Lattke, David A. Donoso, Christoph von Beeren, Michael Heethoff, Sebastian Schmelzle, Adriana Argoti, Luis Camacho, Bernhard Ströbel, Nico Blüthgen

Supplementary material 2 Video of *Odontomachus davidsoni* queen paratype 3D model Data type multimedia Explanation note Rendered video of the 3D scan of the queen paratype. https://binary.pensoft.net/file/431603This dataset is made available under the Open Database License (http://opendatacommons.org/licenses/odbl/1.0/). The Open Database License (ODbL) is a license agreement intended to allow users to freely share, modify, and use this Dataset while maintaining this same freedom for others, provided that the original source and author(s) are credited.Philipp O. Hoenle, John E. Lattke, David A. Donoso, Christoph von Beeren, Michael Heethoff, Sebastian Schmelzle, Adriana Argoti, Luis Camacho, Bernhard Ströbel, Nico Blüthgen

Supplementary material 3 Table with measurements Data type measurements Explanation note Table containing all available measurements of holo- and paratypes of Odontomachus davidsoni. https://binary.pensoft.net/file/431604This dataset is made available under the Open Database License (http://opendatacommons.org/licenses/odbl/1.0/). The Open Database License (ODbL) is a license agreement intended to allow users to freely share, modify, and use this Dataset while maintaining this same freedom for others, provided that the original source and author(s) are credited.Philipp O. Hoenle, John E. Lattke, David A. Donoso, Christoph von Beeren, Michael Heethoff, Sebastian Schmelzle, Adriana Argoti, Luis Camacho, Bernhard Ströbel, Nico Blüthgen

Supplementary material 4 DNA barcode information Data type barcode information Explanation note Table containing a list of the barcoded individuals and their associated collection and repository data. https://binary.pensoft.net/file/431605This dataset is made available under the Open Database License (http://opendatacommons.org/licenses/odbl/1.0/). The Open Database License (ODbL) is a license agreement intended to allow users to freely share, modify, and use this Dataset while maintaining this same freedom for others, provided that the original source and author(s) are credited.Philipp O. Hoenle, John E. Lattke, David A. Donoso, Christoph von Beeren, Michael Heethoff, Sebastian Schmelzle, Adriana Argoti, Luis Camacho, Bernhard Ströbel, Nico Blüthgen

Supplementary material 5 Used BOLD barcodes Data type BOLD accession numbers Explanation note List of BOLD barcode accession numbers used in this study. https://binary.pensoft.net/file/431606This dataset is made available under the Open Database License (http://opendatacommons.org/licenses/odbl/1.0/). The Open Database License (ODbL) is a license agreement intended to allow users to freely share, modify, and use this Dataset while maintaining this same freedom for others, provided that the original source and author(s) are credited.Philipp O. Hoenle, John E. Lattke, David A. Donoso, Christoph von Beeren, Michael Heethoff, Sebastian Schmelzle, Adriana Argoti, Luis Camacho, Bernhard Ströbel, Nico Blüthgen

Supplementary material 6 NJ tree Data type newick tree file Explanation note Newick file of the COI comparisions. https://binary.pensoft.net/file/431607This dataset is made available under the Open Database License (http://opendatacommons.org/licenses/odbl/1.0/). The Open Database License (ODbL) is a license agreement intended to allow users to freely share, modify, and use this Dataset while maintaining this same freedom for others, provided that the original source and author(s) are credited.Philipp O. Hoenle, John E. Lattke, David A. Donoso, Christoph von Beeren, Michael Heethoff, Sebastian Schmelzle, Adriana Argoti, Luis Camacho, Bernhard Ströbel, Nico Blüthgen

Supplementary material 7 Additional detail images of *O. davidsoni* Data type multimedia Explanation note Contains additional detail pictures of the new species. https://binary.pensoft.net/file/431608This dataset is made available under the Open Database License (http://opendatacommons.org/licenses/odbl/1.0/). The Open Database License (ODbL) is a license agreement intended to allow users to freely share, modify, and use this Dataset while maintaining this same freedom for others, provided that the original source and author(s) are credited.Philipp O. Hoenle, John E. Lattke, David A. Donoso, Christoph von Beeren, Michael Heethoff, Sebastian Schmelzle, Adriana Argoti, Luis Camacho, Bernhard Ströbel, Nico Blüthgen

Supplementary material 8 Additional 3D scans of two more paratype workers Data type multimedia Explanation note This file contains two additional 3D scans of two more paratype workers. https://binary.pensoft.net/file/431609This dataset is made available under the Open Database License (http://opendatacommons.org/licenses/odbl/1.0/). The Open Database License (ODbL) is a license agreement intended to allow users to freely share, modify, and use this Dataset while maintaining this same freedom for others, provided that the original source and author(s) are credited.Philipp O. Hoenle, John E. Lattke, David A. Donoso, Christoph von Beeren, Michael Heethoff, Sebastian Schmelzle, Adriana Argoti, Luis Camacho, Bernhard Ströbel, Nico Blüthgen

Supplementary material 9 Table of localities Data type occurence Explanation note Table of known *Odontomachus davidsoni* localities. https://binary.pensoft.net/file/431610This dataset is made available under the Open Database License (http://opendatacommons.org/licenses/odbl/1.0/). The Open Database License (ODbL) is a license agreement intended to allow users to freely share, modify, and use this Dataset while maintaining this same freedom for others, provided that the original source and author(s) are credited.Philipp O. Hoenle, John E. Lattke, David A. Donoso, Christoph von Beeren, Michael Heethoff, Sebastian Schmelzle, Adriana Argoti, Luis Camacho, Bernhard Ströbel, Nico Blüthgen
