# Supplementary figures and images for: Odontomachus davidsoni sp. nov. (Hymenoptera, Formicidae), a new conspicuous trap-jaw ant from Ecuador
Source: Zookeys. 2020 Jul 13;948:75–105. doi: 10.3897/zookeys.948.48701 (PMC7381719; doi:10.3897/zookeys.948.48701)

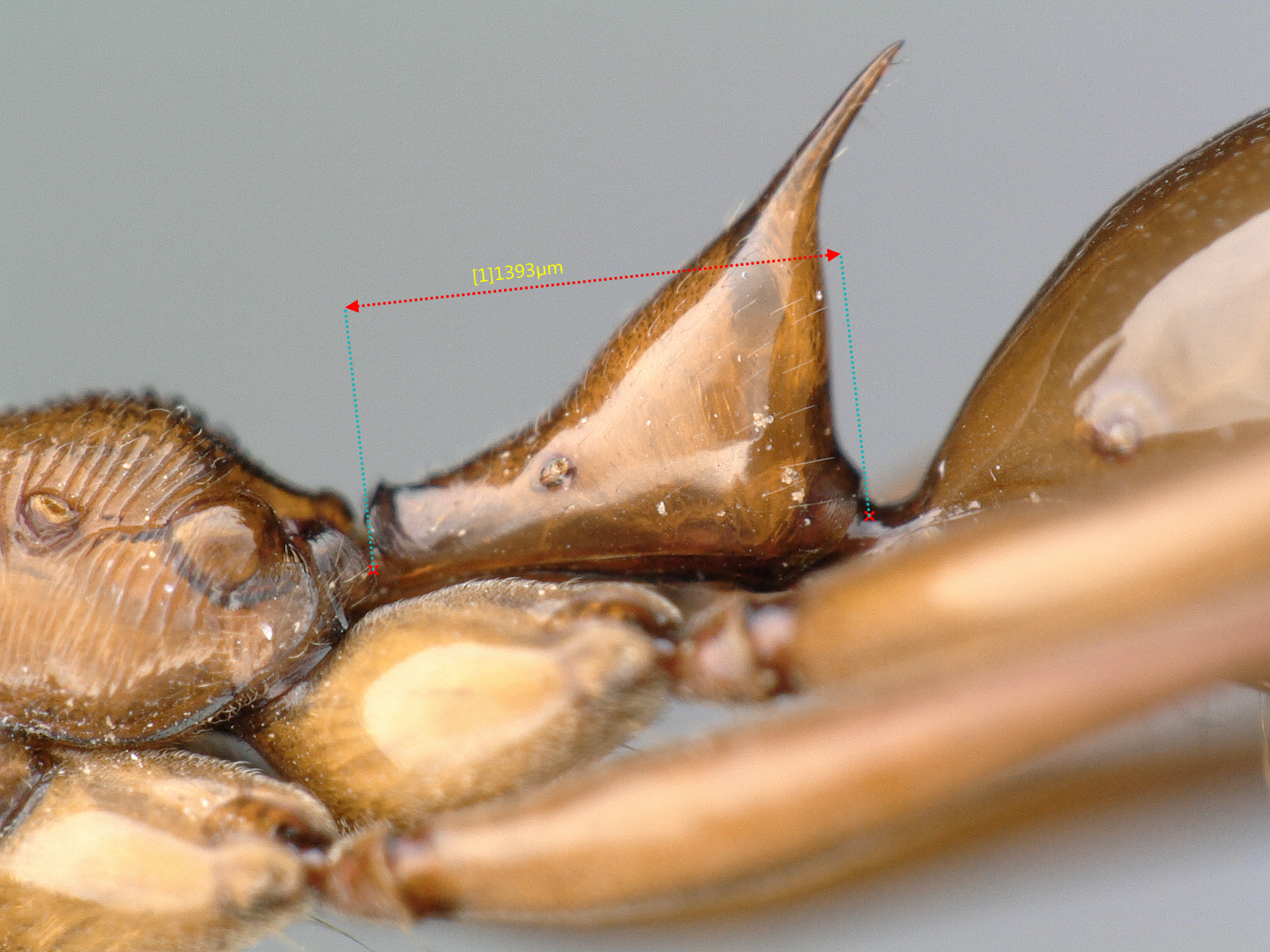

Supplement: Supplementary material 7 — Additional detail images of O. davidsoni [file zookeys-948-075-s007.zip › Supplement file S7 pictures/detail petiole Odon_Phil_3.tif]

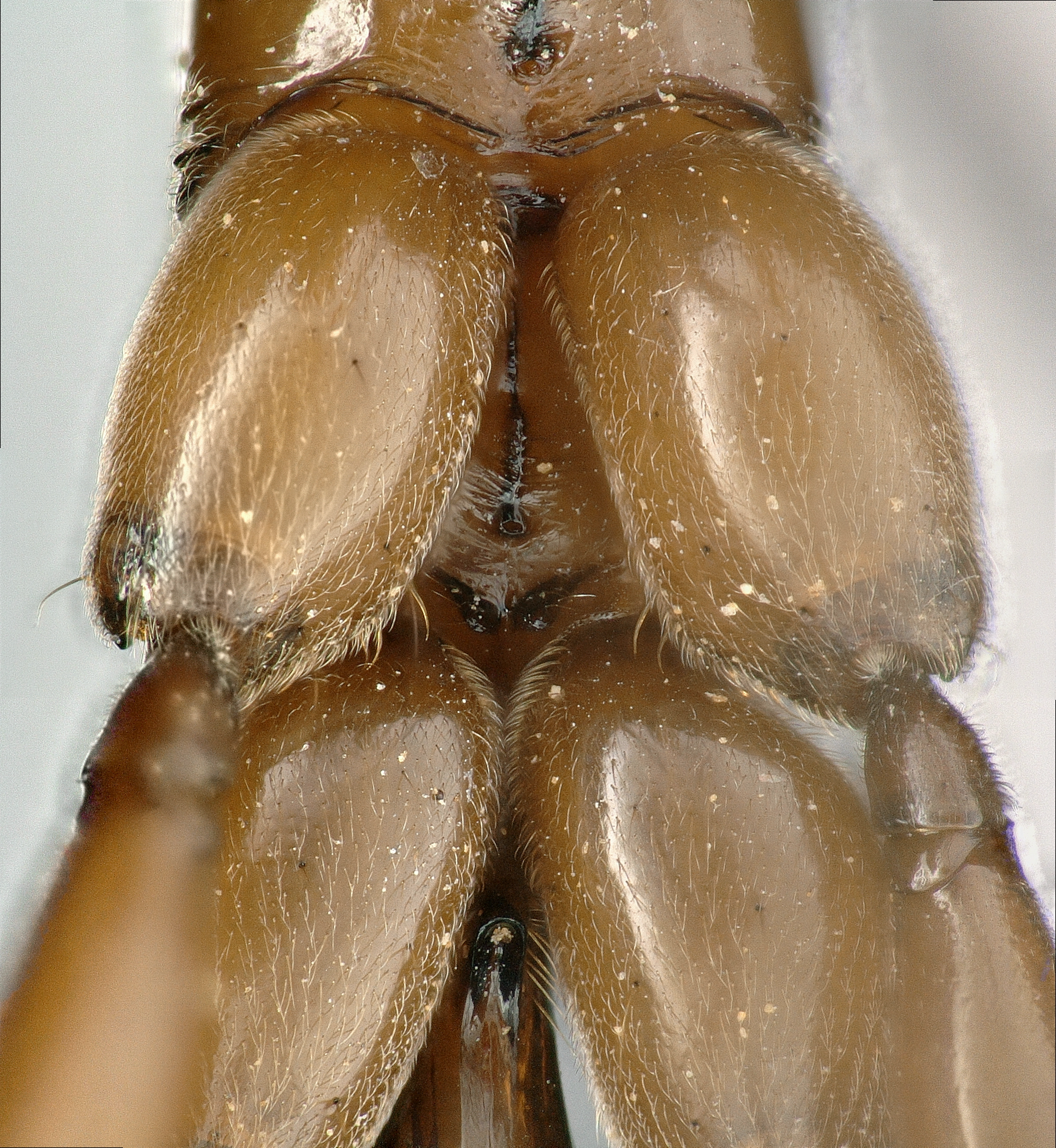

Supplement: Supplementary material 7 — Additional detail images of O. davidsoni [file zookeys-948-075-s007.zip › Supplement file S7 pictures/detail ventral Odon_Phil_3.tif]

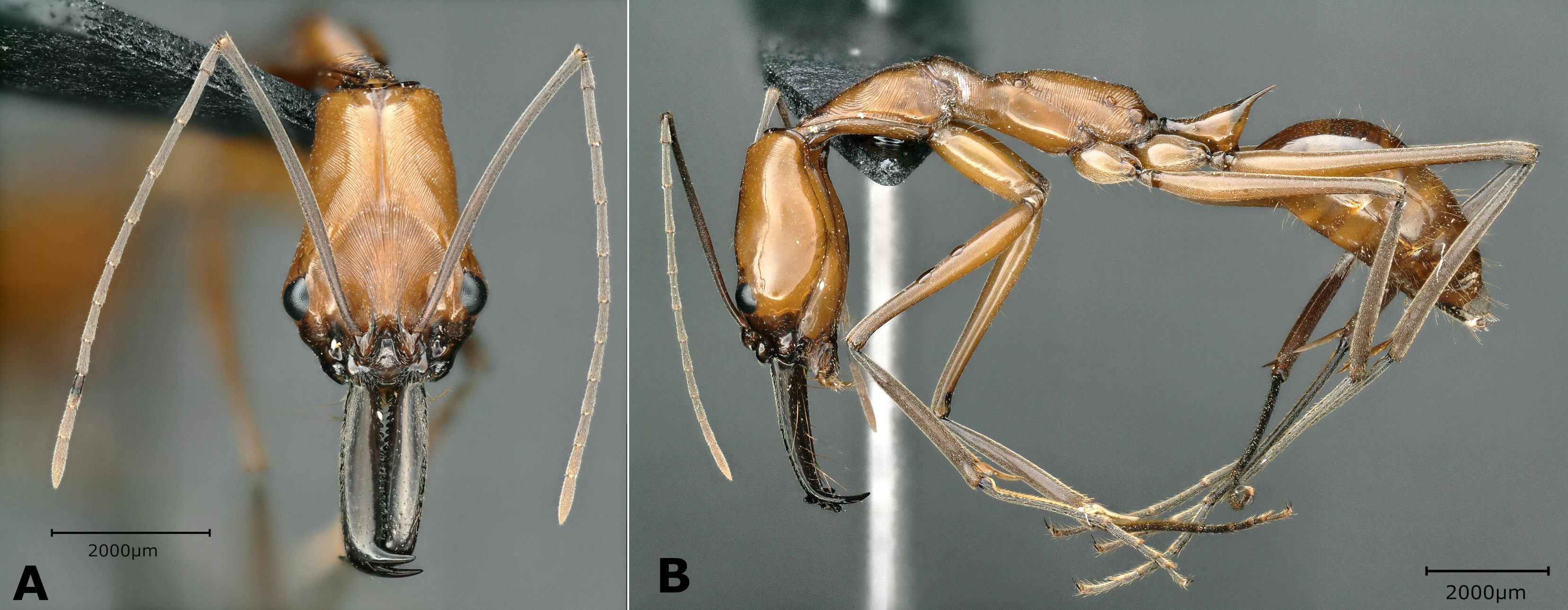

Supplement: Supplementary material 7 — Additional detail images of O. davidsoni [file zookeys-948-075-s007.zip › Supplement file S7 pictures/specimen Odon_Phil_3.jpg]

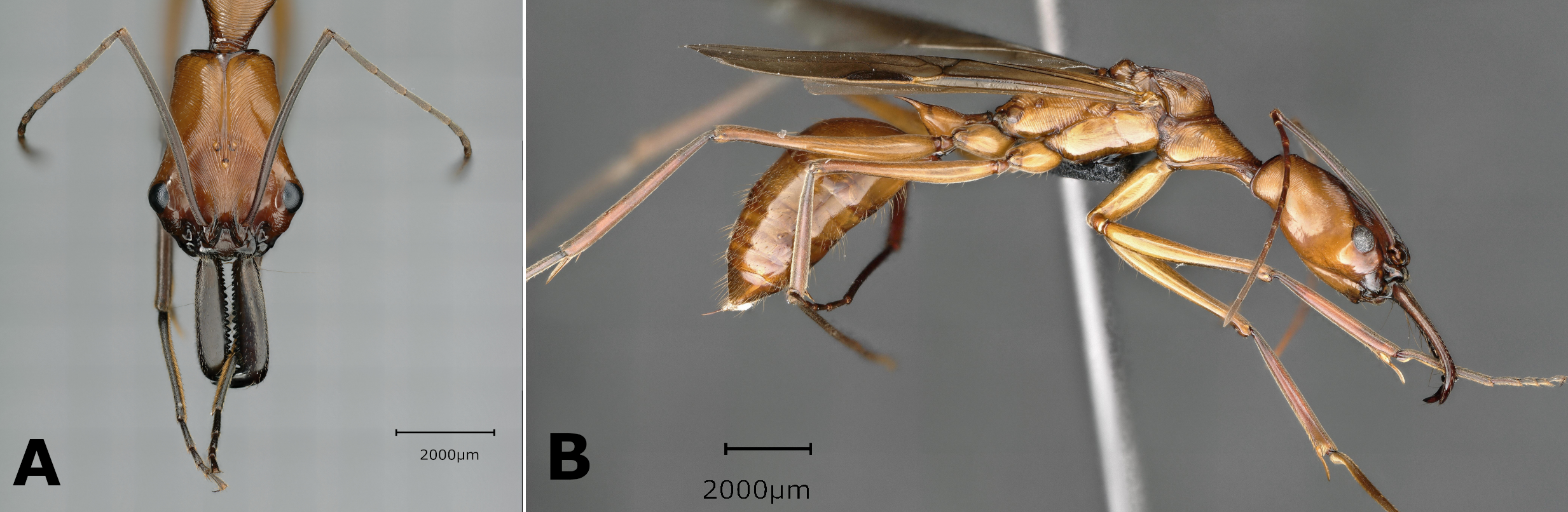

Supplement: Supplementary material 7 — Additional detail images of O. davidsoni [file zookeys-948-075-s007.zip › Supplement file S7 pictures/specimen Odon_Phil_queen.png]
